# Promoting environmentally sustainable food purchases in online grocery shopping: insights from a pilot randomised controlled field trial

**DOI:** 10.1186/s13104-025-07370-5

**Published:** 2025-07-15

**Authors:** Helena Bentil, Oyinlola Oyebode, Peter Scarborough, Claire Thompson, Jessica Brock, Michael Clark, Martin White, Thijs van Rens

**Affiliations:** 1 Department of Economics, University of Warwick, Coventry, UK; 2 Wolfson Institute of Population Health, Queen Mary University of London, London, UK; 3 Nuffield Department of Primary Care Health Sciences, NIHR Oxford Health Biomedical Research Centre, University of Oxford, Oxford, UK; 4 School of Health and Social Work, University of Hertfordshire, Hertfordshire, UK; 5 Smith School of Enterprise and the Environment, University of Oxford, Oxford, UK; 6 Department of Biology, University of Oxford, Oxford, UK; 7 MRC Epidemiology Unit, University of Cambridge, Cambridge, UK

**Keywords:** Eco-labels, Sustainable food purchases, UK, Online grocery shopping

## Abstract

**Objective:**

It remains unclear which interventions are effective in promoting more environmentally sustainable food choices within online grocery shopping environments. We set out to (1) use a plug-in (browser extension) to implement a pilot randomised controlled trial of eco-labels providing information on the environmental impact of specific food products, and (2) collect data to inform a larger trial investigating the effectiveness of eco-labels and other interventions promoting environmentally sustainable online food purchases. The plug-in was custom-built and active on a large UK supermarket website, accessed using the Google Chrome browser on a desktop or laptop.

**Results:**

Of the 504 participants screened, 161 met eligibility criteria and were invited to participate in the study. 57 of these downloaded the plug-in (23 in the control group, 34 in the intervention group), of which 22 shopped at least once over the 1-month trial. There was no significant difference in average eco-score of purchases between the control and intervention groups (mean ± SD: 32 ± 13 vs. 41 ± 14; *p* = 0.22). 69/161 eligible participants responded to a follow-up survey and suggested technical support, reminders, greater incentives, and more information about eco-labels were needed for the full trial. We showed that it is feasible to evaluate online grocery shopping interventions without the collaboration of a supermarket using a web browser extension.

**Trial registration:**

This pilot trial was not registered, as its main purpose was to test the implementation of the plugin and gather data useful for planning the main trial, which is registered under ISRCTN18800054 as of 27/03/2024.

**Supplementary Information:**

The online version contains supplementary material available at 10.1186/s13104-025-07370-5.

## Introduction

The need to meet environmental sustainability targets, such as limiting the global mean temperature rise to under 1.5 °C, has driven efforts to reduce the impacts of the food system, which currently accounts for a third of global greenhouse gas (GHG) emissions [1, 2]. Dietary change is necessary to achieve these environmental targets - improvements in production methods will not be sufficient [3, 4]. Simultaneously, there is a rising prevalence of overweight, obesity, and non-communicable diseases due to poor diet [5, 6]. A global dietary shift towards cereals, fruits, vegetables and pulses could significantly reduce food system emissions from red meat and milk production, as well as prevent up to 11.5 million diet-related deaths per year [7]. As such, encouraging the public to make more sustainable and healthier choices is crucial to reducing GHG emissions and improving health, both of which are high priorities of the UK government.

Online supermarkets provide an opportunity to implement interventions with a broad reach to address these challenges, given the growing popularity of online grocery shopping, which reached 12% of UK sales in 2022 [8]. One potential way to encourage sustainable choices in this environment is with eco-labels that provide information on the environmental impact of foods. Eco-labelling was found to be effective in a systematic review including 56 studies published up to 2019 [9]. However, most included studies were conducted in simulated environments, which are known to potentially overestimate intervention impacts; the few studies conducted in real-world settings did not involve online supermarkets [9].

In this pilot trial, we aimed to (1) use a plug-in (browser extension) to implement a pilot randomised controlled trial of eco-labels to promote more sustainable purchasing and collect food purchase data; and (2) collect data on recruitment, attrition, and the barriers and facilitators to participation among supermarket customers. The results will inform a larger trial investigating the effectiveness of eco-labels and other interventions promoting sustainable food purchases within a real online grocery environment in the UK.

## Method

### Study design and participants

This was a parallel 2-arm pilot randomised controlled trial with a 1:1 participant allocation ratio among shoppers on the website of a UK supermarket in September and October 2023, complemented with online surveys to collect data on socio-demographic characteristics and experiences of participation.

Participants were recruited from the online research platform prolific.com. Participants were eligible for the trial if they lived in the UK, were aged 18 years or over, were the primary grocery shoppers for their household, and indicated that they shop online for groceries at least twice a month. Further, they usually shopped at or were willing to shop at the supermarket that the plug-in was designed for, usually used a desktop or laptop and the Google Chrome browser or were willing to do so for the duration of the study and consented to install the plug-in and keep it enabled when they did their shopping.

Participants were randomised to complete their normal grocery shopping under one of two conditions: with eco-labels visible, providing participants with information on the environmental impact of each food item they viewed online versus no eco-labels. Our plug-in overlaid the eco-labels on the shopping website of the supermarket and collected participants’ grocery purchasing data. The trial had a 2-week baseline data collection phase, during which the eco-labels were ‘off’ for both groups, followed by a 2-week intervention period, during which the eco-labels were turned on for the intervention group.

The study protocol was approved by the University of Warwick’s Humanities & Social Sciences Research Ethics Committee (HSSREC 187/22–23). All participants provided written informed consent before the collection of data. All procedures were conducted in accordance with the ethical standards of the Helsinki Declaration. This study is reported in line with the CONSORT guidelines. This pilot trial was not registered as the main purpose was to test implementation using the plug-in and gather data useful for planning the main trial, which is registered in the BMC ISRCTN registry (ISRCTN18800054).

### Sample size

We invited 500 participants to complete the screening questionnaire with the expectation that this would allow for the recruitment of 100 eligible participants, adequate to meet the study aims. However, we had 161 eligible participants whom we invited to participate in the trial.

### Randomisation and blinding

In the randomisation process, the lead investigator used a random-number generator in Excel to generate two independent uniformly distributed random numbers between 0 and 1 for each participant. We then allocated a participant to the eco-label treatment group if the first random number was smaller or equal to 0.5 and to the no-eco-label group otherwise. Complete participant blinding was not possible due to the visible nature of the eco-labels, and researchers were also not blinded, as group assignments were accessible to the team.

### Intervention

Colour-coded eco-labels providing eco-scores for food and drink products were shown alongside products on the website of a large UK supermarket to the intervention group during the intervention period. Products were given an overall score of 0-100, categorised into seven groups A (best) to G (worst) based on the environmental impact of their constituent ingredients by a commercial company. In brief, our commercial partner (Sustained.com) which also designed the eco-labels and built the plug-in used in this study [10], mapped each food ingredient to life cycle assessment (LCA) databases, such as Agribalyse_v301, and calculated its environmental impacts using OpenLCA software across the 16 categories defined by the Product Environmental Footprint (PEF) methodology [11]. The ingredients were then scored by summing all PEF impacts, with higher scores corresponding to larger environmental impacts and lower scores reflecting more eco-friendly ingredients. Each food product’s impact was calculated using the scores from the ingredients it contained, adjusted by weight.

### Data collection procedures

**Fig. 1:**
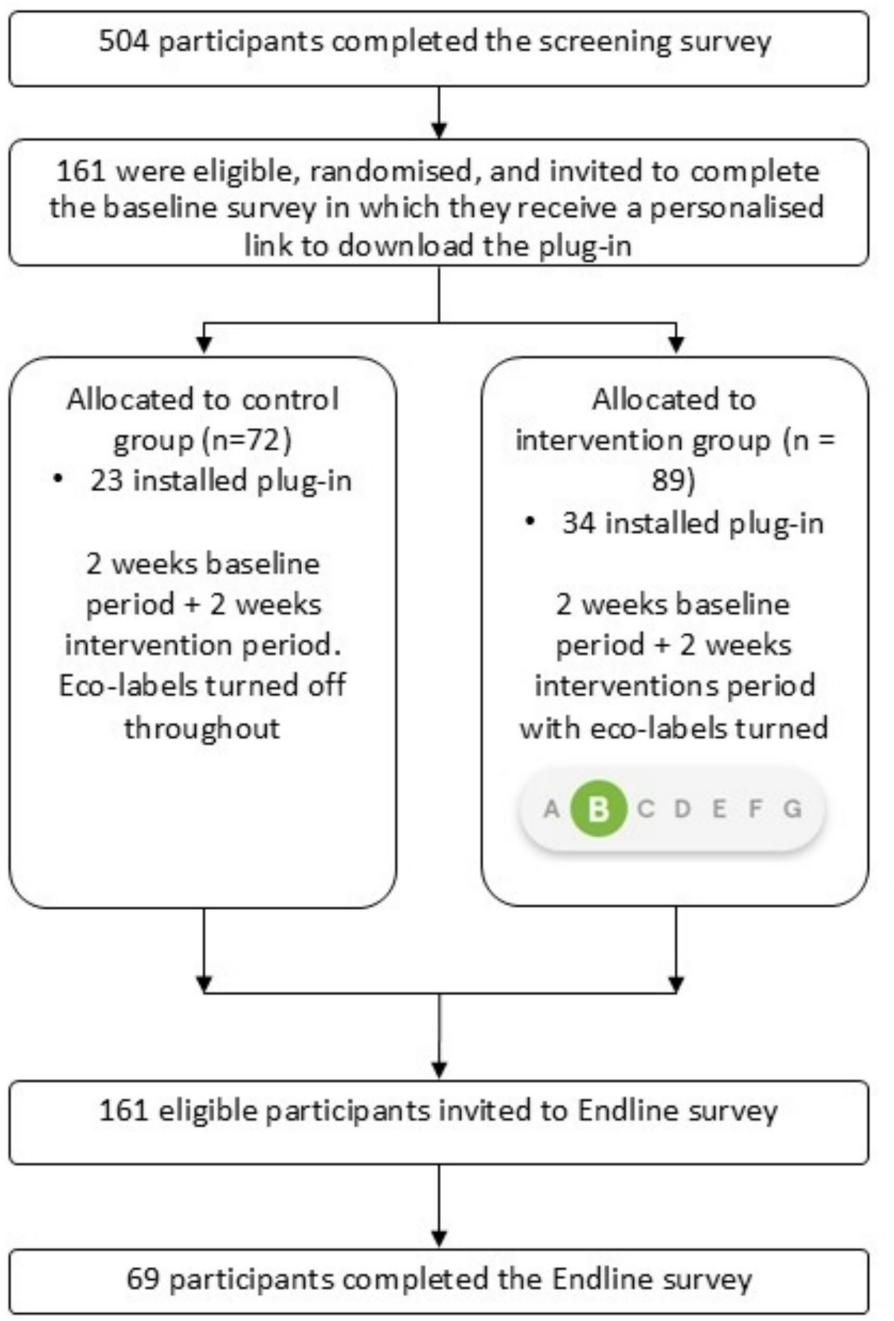
Diagram showing the flow of participants through the data collection process

Participants provided consent and completed a screening survey (**supplementary file**) administered online via Qualtrics to assess eligibility. Following this, eligible participants completed a baseline survey (**supplementary file**) to collect data on socio-demographic characteristics and received instructions and a customised download link to the plug-in. After installing the plug-in, participants were asked to complete their normal online grocery shopping on the supermarket website over 4 weeks. During this period, the plug-in collected data on participants’ grocery purchases. At the end of the intervention period, participants completed another online survey (the follow-up survey, **supplementary file**), providing quantitative and qualitative data about their experiences of participation, using the plug-in, and the eco-label intervention. Participants were reimbursed for completing online surveys and received £5 compensation for completing at least one shop with the plug-in installed during the 4-week study duration. Figure 1 illustrates the study design and participant flow.

### Data analysis

Descriptive statistics were used to examine socio-demographics, participation rates, attrition rates and experiences of participation recorded in the follow-up survey. Free-text responses to the follow-up survey were analysed using qualitative content analysis, with themes identified inductively through close reading of the data in Excel. Differences in the mean eco-score of baskets between groups (based on the same methodology as the scores on the eco-labels) were checked with t-tests. Statistical analysis was conducted using STATA 18 (StataCorp LLC, TX, USA) and qualitative analysis in Excel.

## Results

Using a custom-built plug-in we were able to implement a pilot randomised controlled field trial in which eco-labels were displayed to intervention participants and data were collected on their purchasing behaviour, which could be matched to participant data from online surveys.

The 504 Prolific participants screened were similar in terms of gender composition, but younger (*p* < 0.001) and more highly educated (*p* < 0.001) than the population average (Table 1). Out of the 504, 161 were found eligible and invited to join the study. The eligible and invited sample were younger (*p* = 0.039) and had higher income (*p* < 0.001) compared with the screened participants who were ineligible. Fifty-seven of those invited installed the plug-in. Those who installed the plug-in were more educated (*p* = 0.032) and had higher average income (*p* < 0.001) than those who were eligible and invited but did not install the plug-in, but the groups were similar in terms of age and ethnicity. Both eligible and invited participants, as well as those who installed the plug-in, were mostly women, aged 18–59, with at least an undergraduate degree or higher, and predominantly identified as white ethnicity. Of the 57 participants who installed the plug-in, only 22 participants shopped at least once over the 1-month trial period (10 shopped once and 12 shopped twice or more).

**Table 1:** Summary statistics of study participants

	UK Census 2021 %	Characteristics of study participants			Eco-basket score^#^ Mean ± SD	Number of purchased products Mean ± SD	Total cost (£) Mean ± SD
		Screening survey *n* (%)	Eligible and invited^$^ *n* (%) / Mean ± SD	Installed plug-in *n* (%)/ Mean ± SD			
Total participants, N		504	161 (32%)	57 (35%)	36.06 ± 25.45	28.52 ± 19.26	71.27 ± 47.86
Gender							
Women	51%	243 (48%)	75 (47%)	28 (49%)	40.42 ± 27.31	28.29 ± 19.59	76.47 ± 49.59
Men	49%	261 (52%)	86 (53%)	29 (51%)	30.17 ± 21.93	28.83 ± 19.24	64.26 ± 45.56
Educational level							
<= Tech/comm college	66%	208 (42%)	60 (37%)	16 (28%)	31.09 ± 19.01	26.64 ± 18.06	56.49 ± 36.93
>= Undergrad degree	34%	296 (58%)	101 (63%)	41 (72%)	39.47 ± 28.87	29.81 ± 20.22	81.43 ± 52.26
Age Group							
18–59	55%	430 (85%)	145 (90%)	48 (84%)	30.33 ± 20.91	30.52 ± 21.94	69.03 ± 52.60
60–84	22%	74 (15%)	16 (10%)	9 (16%)	50.93 ± 30.61	23.27 ± 7.54	77.12 ± 33.36
Ethnicity							
White	82%	-	61 (78%)	44 (77%)	36.68 ± 25.95	26.40 ± 15.99	66.12 ± 40.10
Other	18%	-	16 (22%)	13 (23%)	28.25 ± 18.91	55.00 ± 36.94	136.71 ± 90.71
Lone parent household⁺	11%	-	22 (28%)	11 (19%)	37.50 ± 26.99	26.00 ± 17.72	50.76 ± 28.84
Average Income^‡^	£37,622	-	£33,130.55 ± £23,251.66	30,604.08 ± £20,648.02	-		
<= £20,000	-	-	26 (16%)	21 (37%)	21.83 ± 22.86	21.83 ± 22.86	40.85 ± 35.99
> £20,000	-	-	135 (84%)	36 (63%)	43.17 ± 23.90	32.44 ± 17.75	86.48 ± 46.11

⁺ Lone-parent household - A household with a single adult living with children

^‡^ Per-person equivalent income, calculated from household income and composition by the researchers

^$^ There were actually 173 eligible but due to human error not all of these were invited to install the plug-in and participate in the pilot trial

^#^ Scores are between 0 and 100; A lower score is more sustainable

The 161 eligible participants invited to join the study were also invited to complete the follow-up survey. Out of these, 69 responded. Most participants who responded to the follow-up survey reported installing the plug-in (61/69), with 4 of the 61 misreporting, as only 57 had actually installed the plug-in. Of the participants who reported installing the plug-in, most reported that they did not have problems with it (59/61) (Table 2). Those who did not install it gave the following reasons: did not know how to install the plug-in, mistrust, and corrupted plug-in. Most participants who did install the plug-in reported that they had not uninstalled it at the time of the follow-up survey (51/59). Those who uninstalled it cited mistrust and security concerns as the reasons, or because the study was over without specifying a further reason. Most participants who reported that they did not uninstall the plug-in indicated that they were not planning to because they liked the information that the plug-in gave them and would continue to use it (Table 2).

**Table 2:** Follow-up survey results^1^

	No	Yes	*n* (%)
Did you install the plug-in (*n* = 69)	8 (12%)	61 (88%)	
Did you have any problems with the plug-in (*n* = 61)	59 (97%)	2 (3%)	
Did you uninstall the plug-in? (*N* = 59)	51 (86%)	8 (14%)	
Are you planning to uninstall the plug-in? (*N* = 51)	31 (61%)	20 (39%)	
Did you notice the eco-labels? (*N* = 20)	0 (0%)	20 (100%)	
Why do you not plan to uninstall the plug-in? (*N* = 30)			
I quite like the information that the extension gives me and will continue to use it			17 (58%)
I will use a different supermarket website or a different device for online grocery shopping in the future			3 (10%)
I don’t use the information, but it doesn’t bother me either			10 (32%)
Did you consider the eco-labels when making grocery shopping decisions (*n* = 20)	7 (14%)	13 (86%)	
Did the eco-labels make you change your mind about what to buy? (*N* = 13)	5 (38%)	8 (62%)	
If you bought something different because of seeing the eco-labels, will you buy these different products again in the future? (*N* = 8)	1 (12%)	7 (88%)	
How much of your household grocery shopping was completed at the supermarket on Chrome? (*N* = 59)			
All of my household grocery shopping			16 (27%)
Most of my household grocery shopping			12 (20%)
Some of my household grocery shopping			11 (19%)
A little of my household grocery shopping			5 (9%)
None of my household grocery shopping			15 (25%)

^1^161 eligible participants were invited, of which 69 responded to the follow-up survey. The number of respondents for each question varies based on the skip logic of the survey depending on the response

Of 59 follow-up survey participants who reported installing the plug-in, 43 reported doing some or none of their household grocery shopping at the online supermarket rather than all, as requested (Table 2), because they forgot, did not have the time, had to go to the shop in person, or because other household members did the shopping or for supermarket-specific reasons like costly products, product unavailability, or having a loyalty card at a different supermarket.

All 20 participants in the intervention group who responded to the follow-up survey reported noticing the eco-labels. All 20 reported they were easy to understand, and 13 of these used the eco-labels when deciding what to buy. Eight participants reported that the eco-labels made them change their minds about what to buy, with seven of these indicating they would purchase the items they had switched to again in the future (Table 2). Those who reported that the eco-labels did not influence their purchasing said they were too busy; wanted to stick with what they usually buy; needed the unsustainable items; were already buying sustainable products; mistrusted the labels; or found the sustainable foods too expensive.

The average (± standard deviation) eco-score of the basket of products purchased in the intervention period was 32 ± 13 in the control group and 41 ± 14 in the intervention group. As expected, as this was a pilot trial, the difference of 9.5 was not significant, with a p-value of 0.22. The difference in the mean change from baseline to intervention periods was also not significantly different (*p* = 0.66, -4 ± 2 in the control group, -14 ± 42 in the intervention group). The longitudinal estimates were less precise because number of participants that shopped at least once in the baseline period as well as at least once in the intervention period was much lower, and the standard deviation in basket eco-scores within individual participants (16.2) was almost as large as the one between participants (16.4).

## Discussion

Our pilot trial on the use of eco-labels to promote environmentally sustainable choices in online grocery shopping, using a plug-in on the website of a real supermarket, showed the feasibility of the approach. We gathered valuable insights and preliminary findings that will improve the design and implementation of the larger-scale trial.

The percentage of eligible participants who downloaded the plug-in was lower than expected (35%). Our follow-up survey revealed that participants did not trust the plug-in due to security concerns, and some reported they did not know how to install it. To address these concerns and increase the percentage of people who download plug-ins for research it may help for participants to have access to additional information (e.g. ethics approval documents and information on data protection) and links to the affiliated institutions and online profiles of the researchers involved, confirming the project’s legitimacy. Support for those with technical difficulties could be provided by videos offering step-by-step instructions.

To respond to participants’ queries about the legitimacy of the eco-label scores in our larger trial, we have developed information on the study webpage [10] to help future participants understand how the eco-score is calculated and why a product’s eco-score may be lower or higher than expected. If participants believe a product’s eco-score is incorrect after reading the information available, they can report this via the plug-in, and our team will review and provide feedback.

The percentage of participants who shopped throughout this study was lower than expected. This may have been partly because of the relatively short intervention period. Additionally, some participants revealed that they forgot to complete their regular shopping with the plug-in or that other household members had completed their shopping during the study duration. Those who do not usually shop at the supermarket used in this study gave many reasons for not completing their shopping with the plug-in. We anticipate that addressing these issues would help increase the percentage of people who shop using a plug-in in research studies. In the larger trial, we will have a longer duration and will not include a baseline period, as our pilot showed that there is no efficiency gain from focusing on intra-participant variation in purchases. In addition, we will not recruit participants who usually shop at other supermarkets. We will send regular reminders to participants to shop using the plug-in, and if they have not already downloaded it, to do so. We will offer future participants the option of downloading the plug-in onto an additional desktop/laptop within the same household. We will also increase compensation to appropriately reward participants for their time and effort.

### Limitations

Participants were recruited from an online research agency on a Monday morning. The study was advertised at 8 am and by 8.03 we had reached our intended participant numbers. In addition, the trial required the use of a laptop/desktop. For these reasons, the sample used in this study probably excluded certain demographic groups, for example, those who are more likely to use mobile phones or tablets for online shopping are unlikely to represent the general population of UK online grocery shoppers.

## Electronic supplementary material

Below is the link to the electronic supplementary material.

Supplementary Material 1

Supplementary Material 2

## Data Availability

Data can be provided upon request.

## References

[1] Smith P, Clark H, Dong H, Elsiddig EA, Haberl H, Harper R, House J, Jafari M, Masera O, Mbow C, Ravindranath NH, Rice CW. Roble do Abad, C., Romanovskaya, A., Sperling, F., & Tubiello, F. (2014). Chapter 11 - Agriculture, forestry and other land use (AFOLU). http://www.ipcc.ch/pdf/assessment-report/ar5/wg3/ipcc_wg3_ar5_chapter11.pdf

[2] Crippa M, Solazzo E, Guizzardi D, Monforti-Ferrario F, Tubiello FN, Leip A. Food systems are responsible for a third of global anthropogenic GHG emissions. Nat Food 2021. 2021;2:3(3):198–209. 10.1038/s43016-021-00225-9. 10.1038/s43016-021-00225-9 37117443

[3] Springmann M, Clark M, Mason-D’Croz D, et al. Options for keeping the food system within environmental limits. Nature. 2018;562:519–25. 10.1038/s41586-018-0594-0. 30305731 10.1038/s41586-018-0594-0

[4] Clark MA, Domingo NGG, Colgan K, Thakrar SK, Tilman D, Lynch J, Azevedo IL, Hill JD. Global food system emissions could preclude achieving the 1.5° and 2°C climate change targets. Science. 2020;370(6517):705–708. 10.1126/science.aba7357. PMID: 33154139. 10.1126/science.aba7357 33154139

[5] Ryan D, Barquera S, Barata Cavalcanti O, Ralston J. The global pandemic of overweight and obesity. In: Kickbusch I, Ganten D, Moeti M, editors. Handbook of global health. Cham: Springer; 2021. 10.1007/978-3-030-45009-0_39.

[6] Palmer D. Estimating the full costs of obesity. Frontier Economics: A report for Novo Nordisk; 2022.

[7] Romanello M, Di Napoli C, Drummond P, Green C, Kennard H, Lampard P, Scamman D, Arnell N, Ayeb-Karlsson S, Ford LB, Belesova K, Bowen K, Cai W, Callaghan M, Campbell-Lendrum D, Chambers J, van Daalen KR, Dalin C, Dasandi N, Dasgupta S, Davies M, Dominguez-Salas P, Dubrow R, Ebi KL, Eckelman M, Ekins P, Escobar LE, Georgeson L, Graham H, Gunther SH, Hamilton I, Hang Y, Hänninen R, Hartinger S, He K, Hess JJ, Hsu SC, Jankin S, Jamart L, Jay O, Kelman I, Kiesewetter G, Kinney P, Kjellstrom T, Kniveton D, Lee JKW, Lemke B, Liu Y, Liu Z, Lott M, Batista ML, Lowe R, MacGuire F, Sewe MO, Martinez-Urtaza J, Maslin M, McAllister L, McGushin A, McMichael C, Mi Z, Milner J, Minor K, Minx JC, Mohajeri N, Moradi-Lakeh M, Morrissey K, Munzert S, Murray KA, Neville T, Nilsson M, Obradovich N, O’Hare MB, Oreszczyn T, Otto M, Owfi F, Pearman O, Rabbaniha M, Robinson EJZ, Rocklöv J, Salas RN, Semenza JC, Sherman JD, Shi L, Shumake-Guillemot J, Silbert G, Sofiev M, Springmann M, Stowell J, Tabatabaei M, Taylor J, Triñanes J, Wagner F, Wilkinson P, Winning M, Yglesias-González M, Zhang S, Gong P, Montgomery H, Costello A. The 2022 report of the Lancet Countdown on health and climate change: health at the mercy of fossil fuels. Lancet. 2022;400(10363):1619–1654. 10.1016/S0140-6736(22)01540-9. Epub 2022 Oct 25. Erratum in: Lancet. 2022;: Erratum in: Lancet. 2022;400(10365):1766. PMID: 36306815.

[8] Statista. (2023). Online grocery shopping in the United Kingdom (UK) - statistics and facts. Retrieved from https://www.statista.com/topics/3144/online-grocery-shopping-in-the-united-kingdom/#topicOverview. Published by Statista Research Department on Dec 19, 2023.

[9] Potter C, Bastounis A, Hartmann-Boyce J, Stewart C, Frie K, Tudor K, Bianchi F, Cartwright E, Cook B, Rayner M, Jebb SA. The effects of environmental sustainability labels on selection, purchase, and consumption of food and drink products: A systematic review. Environ Behav. 2021;53(8):891–925. Epub 2021 Feb 20. PMID: 34456340; PMCID: PMC8384304. 34456340 10.1177/0013916521995473 PMC8384304

[10] Eco-labels (n.d). The Sustained Trial. Retrieved July 04. 2024, from https://www.salientfoodtrials.uk/trials/sustained_folder/participants/participants

[11] European Commission: Joint Research Centre, Damiani, M., Ferrara, N. and Ardente, F., Understanding Product Environmental Footprint and Organisation Environmental Footprint methods, Publications Office of the European Union. 2022, https://data.europa.eu/doi/10.2760/11564

