# Supplementary material for: Promoting environmentally sustainable food purchases in online grocery shopping: insights from a pilot randomised controlled field trial
Source: BMC Res Notes. 2025 Jul 15;18:294. doi: 10.1186/s13104-025-07370-5 (PMC12265105; doi:10.1186/s13104-025-07370-5)
Supplement: Supplementary file 1 — Supplementary Material 1 [file 13104_2025_7370_MOESM1_ESM.pdf]

Screening questionnaire

We would like to ask some questions about whether you do your grocery shopping online, and if so how.

0. [Prolific screener question] Are you the main (or shared) grocery shopper for the food that your household eats?
   - Yes
   - No *(If no, participants will not be invited to take this survey)*

1. What gender are you currently?
   - Man (including Trans Male/Trans Man)
   - Woman (including Trans Female/Trans Woman)
   - Non-binary (would like to give more detail)
   - Rather not say

2. What is your date of birth? *(All participants on Prolific are 18+)*
   - _______________
   - Rather not say

3. Which of these is the highest level of education you have completed?
   - No formal qualifications
   - Secondary education (e.g. GED/GCSE)
   - High school diploma/A-levels
   - Technical/community college
   - Undergraduate degree (BA/BSc/other)
   - Graduate degree (MA/MSc/MPhil/other)
   - Doctorate degree (PhD/other)
   - Don't know / not applicable

4. In which postcode area do you live?

5. Do you ever buy groceries online?
   - Yes
   - No *(If no, end the survey here)*

6. How frequently do you buy groceries online?
   - Less than once a month
   - About once a month
   - About twice a month
   - About once a week
   - More than once a week

7. Which supermarket do you use most often for your online grocery shopping?
   - Tesco
   - Sainsbury's
   - Asda

- Morrisons
- Aldi
- Ocado
- Amazon
- Other, please specify ____________________

8. Would you be willing to do your grocery shopping at a different online grocery shop to participate in a research study?
   - Yes
   - No *(If no, end the survey here)*

9. Would you be willing to do your online shopping at the study's supermarket for the duration of a 4-week study?
   - Yes
   - No *(If no, end the survey here)*

10. What device do you usually use for online grocery shopping?
    - Desktop *(If Desktop go to Q12)*
    - Laptop *(If Laptop go to Q12)*
    - Tablet / iPad
    - Mobile phone
    - Other, please specify ____________________

11. Do you use the store's app or their website?
    - App *(If App go to Q13)*
    - Website

12. Which browser do you use? (Which of the following apps do you click on to access the internet?)
    - Google Chrome *(If Google Chrome go to Q14)*
    - Safari
    - Microsoft Edge
    - Mozilla Firefox
    - Opera
    - Other, please specify __________________

13. Would you be willing to use Google Chrome instead of your usual app or browser for the duration of a 4-week study?
    - Yes
    - No *(If no, end survey here)*

14. Would you be willing to take part in a study about online grocery shopping that requires you to install some software on your computer? The software (a browser extension or "plug in" for Chrome) will give you more information about different food products while you shop and will collect data on your grocery purchases.
    - Yes, I am in principle willing to participate
      (We will contact you through Prolific if you are eligible)
    - No, I would not want to participate in this study

Baseline survey

1. Including you, how many people live in your household?

2. How many people of different ages live in the same house and share groceries with you? (please enter 0 if the answer to any category is none)

Infants and small children (0-4 years old)	
Children (5-11 years old)	
Young people(12-17 years old)	
Adults (18-64 years old)	
Older people (65 years and over)	

3. What is your total household income per year, including all earners in your household (after tax) in GBP?
   - Less than £10,000
   - £10,000 - £15,999
   - £16,000 - £19,999
   - £20,000 - £29,999
   - £30,000 - £39,999
   - £40,000 - £49,999
   - £50,000 - £59,999
   - £60,000 - £69,999
   - £70,000 - £79,999
   - £80,000 - £89,999
   - £90,000 - £99,999
   - £100,000 - £149,999
   - More than £150,000
   - Rather not say

4. What ethnic group do you belong to?
   - White
   - Black
   - Asian
   - Mixed
   - Other

5. Download instructions

Thank you for answering the above questions. Now you are ready to get started with the project. Please carefully read these instructions on what to do next.

Step 1. Download the extension

Click on the download link below to install the browser extension. The extension will only be active at the study's supermarket, and we will not collect any data about you other than about the groceries you buy.

Click here to download

Step 2. Register the extension

Open **the supermarket's website** in Chrome, like you were going to do your online grocery shopping.

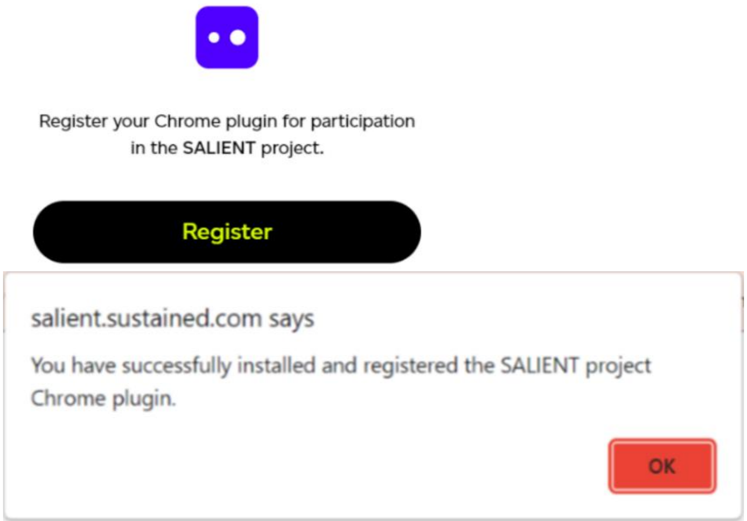

You should see a **Register** button in the bottom-right corner of the screen. Please **click this button**. You should then see a pop-up window confirming that you have successfully registered. Click OK.

Step 3. Check that the installation was successful

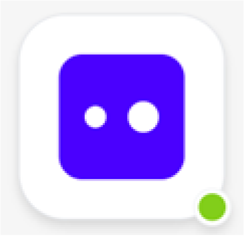

To make sure that the extension is installed correctly, you should look for the Sustained logo at the bottom-right corner of your screen every time you visit the supermarket website. If you do not see the logo immediately, you may need to refresh the page.

Step 4. Return to this page to submit this survey

Do not click the next button until you have successfully installed the browser extension. Once you have completed all steps above, click next to register your completion.

If you have any problem with the installation of the extension, please get in touch with us at sustainedfoodtrial@warwick.ac.uk and we will help you.

Endline survey

These questions help us understand whether the study worked well and what could be improved in the future.

1. Did you install the browser extension?
- Yes *(Go to question 3)*
- No

2. Why not? (free text) *(Go to question 24)*

3. Did you have any problems with the browser extension?
- Yes
- No *(Go to question 5)*

4. What problems did you have? (free text)

5. Did you uninstall the browser extension?
- Yes
- No *(Go to question 7)*

6. Why? (free text)

7. Are you planning to uninstall the browser extension?
- Yes
- No *(Go to question 9)*

8. Why? (free text) *(Go to question 10)*

9. Why is that?
- I quite like the information that the extension gives me and will continue to use it
- I don't use the information, but it doesn't bother me either
- I will use a different supermarket website or a different device for online grocery shopping in the future
- I would like to uninstall it, but I don't know how
- Other, please specify ______________________

10. How much of your household grocery shopping was completed at the supermarket on Chrome while the browser extension was running during the 4-week study?
- All of my household grocery shopping *(Go to question 12)*
- Most of my household grocery shopping
- Some of my household grocery shopping
- A little of my household grocery shopping
- None of my household grocery shopping

11. What were the reasons that you did not complete all of your household grocery shopping at the supermarket with the browser extension? (free text)

12. *Intervention group only:* Did you notice the eco-labels that appeared after [DATE]?

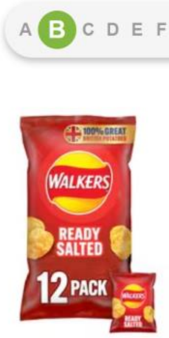

- Yes
- No *(Go to question 19)*

13. *Intervention group only:* Did you find the eco-labels easy to understand and useful?

- Yes, both understandable and useful *(Go to question 14)*
- Easy to understand, but not useful *(Go to question 15)*
- Useful, but not easy to understand *(Go to question 16)*
- No, they are confusing and not useful *(Go to question 16)*

14. Anything we could do to make the labels even better? (free text) *(Go to question 17)*

15. What else would have been more useful to you? (free text) *(Go to question 17)*

16. What would have helped you to make sense of the labels? (free text) *(Go to question 17)*

17. Which of the following two snacks is more sustainable (less damaging to the environment)?

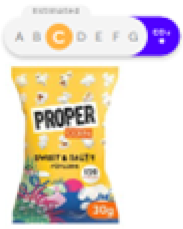 Propercorn Sweet & Salty 30G	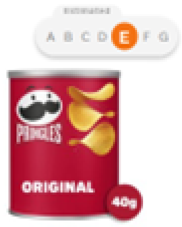 Pringles Pop & Go Original 40G

- The popcorn
- The Pringles
- Their environmental impact is very similar
- I don't know

18. *Intervention group only:* Did you consider the eco-labels when making decisions about what to buy?

- Yes *(Go to question 20)*
- No

19. Why not? (free text)

20. Did the eco-labels make you change your mind about what to buy?

- Yes *(Go to question 22)*
- No

21. Why not? (free text) *(Go to question 24)*

22. Could you tell us what you changed? (free text)

23. If you bought something different because of seeing the eco-labels, will you buy these different products again in the future?

- Yes
- No
- I didn't change what I bought

24. Is there anything else you would like to tell us about online shopping while using the plug-in or anything else about your experience of taking part in this study? (free text)

25. Are you willing to participate in a face-to-face video call?

- Yes, I would be willing to take part in a face-to-face video interview
- Yes, I would be willing to take part in a non-video interview
- Yes, I would be willing to take part in a video focus group
- Yes, I would be willing to take part in a non-video focus group
- No, I would not be willing to take part in an interview or focus group *(If no, end survey here)*

26. We would like to invite you to take part in a short video or audio call interview with one of our researchers about your experience in this study. We will compensate you for your time at the same hourly rate as for this survey (£12/hr). If you are interested in participating, you can schedule the interview at a time that suits you. [Add link to Calendly or similar scheduling tool] To maintain your anonymity, please use your Prolific email address (your participant ID followed by @email.prolific.co) to sign up.
